# Genome-Wide Identification and Expression Patterns of the C2H2-Zinc Finger Gene Family Related to Stress Responses and Catechins Accumulation in *Camellia sinensis* [L.] O. Kuntze

**DOI:** 10.3390/ijms22084197

**Published:** 2021-04-18

**Authors:** Shiyang Zhang, Junjie Liu, Guixian Zhong, Bo Wang

**Affiliations:** The Key Laboratory of Plant Molecular Breeding of Guangdong Province, College of Agriculture, South China Agricultural University, Guangzhou 510642, China; zhangshiyang1997@126.com (S.Z.); junjie_liu202@163.com (J.L.); zhongguixian2021@163.com (G.Z.)

**Keywords:** tea, C2H2-zinc finger gene family, phylogenetic analysis, stress responses, catechins accumulation

## Abstract

The C2H2-zinc finger protein (C2H2-ZFP) is essential for the regulation of plant development and widely responsive to diverse stresses including drought, cold and salt stress, further affecting the late flavonoid accumulation in higher plants. Tea is known as a popular beverage worldwide and its quality is greatly dependent on the physiological status and growing environment of the tea plant. To date, the understanding of C2H2-ZFP gene family in *Camellia sinensis* [L.] O. Kuntze is not yet available. In the present study, 134 Cs*C2H2-ZFP* genes were identified and randomly distributed on 15 chromosomes. The CsC2H2-ZFP gene family was classified into four clades and gene structures and motif compositions of CsC2H2-ZFPs were similar within the same clade. Segmental duplication and negative selection were the main forces driving the expansion of the CsC2H2-ZFP gene family. Expression patterns suggested that *CsC2H2-ZFPs* were responsive to different stresses including drought, salt, cold and methyl jasmonate (MeJA) treatment. Specially, several *C2H2-ZFPs* showed a significant correlation with the catechins content and responded to the MeJA treatment, which might contribute to the tea quality and specialized astringent taste. This study will lay the foundations for further research of C2H2-type zinc finger proteins on the stress responses and quality-related metabolites accumulation in *C. sinensis*.

## 1. Introduction

As the abundant transcriptional regulator in higher plants, the cysteine-2/histidine-2 (C2H2)-type zinc finger protein family (C2H2-ZFP) is composed of two cysteines and two histidines in a conserved sequence motif (CX_2–4_CX_3_PX_5_LX_2_HX_3–5_H) [1,2]. The zinc atom binds to the conserved amino acids of C2H2-ZFP to form a compact structure that binds with the major groove of B-DNA and wraps partway around the double helix in a sequence-specific manner [3]. C2H2-ZFPs perform their function during the various stages of plant development, including trichome initiation and branching, floral organ development, and seed coat development, etc. ZFP8 (AT2G41940), GIS (AT3G58070) and GIS2 (AT5G06650) have been identified as essential regulators of trichome initiation and branching by mediating *GLABROUS1* (*GL1*) expression in response to gibberellin and cytokinin in *Arabidopsis thaliana* (L.) Heynh [4,5]. GIS controls the trichome cell division and negatively regulates trichome branching during trichome development either through directly acting on the negative regulator of gibberellic acid signaling SPINDLY or through indirectly interacting genetically with a key endoreduplication regulator SIAMESE [5,6]. In addition, SGR5 (At2g01940) and SUP (At3g23130) are involved in the inflorescence stems gravitropism and floral meristem development, respectively [7,8]. The C2H2-ZFP, POPOVICH, is crucial to the development of floral nectar spurs in the columbine genus *Aquilegia* [9]. TRANSPARENT TESTA 1 (At1g34790, TT1) is involved in the differentiation of seed endothelium [10]. Interestingly, TT1 further restores pigment accumulation in the endothelium of *Arabidopsis tt1* mutant seeds [11]. Previous studies have showed that *Arabidopsis* C2H2-ZFP TT1 interacts with both CHS, encoding the first enzyme of the catechins biosynthetic pathway, and the R2R3 MYB protein TT2, regulating the late steps of catechins biosynthesis [11]. The ectopic expression of *TT2* can partially restore the lack of proanthocyanidin pigment production in *tt1* mutant, which is polymerized by catechins [11,12]. Furthermore, silencing of *BnTT1* genes, homologous copies of *TT1*, causes reduced seed epicatechin accumulation in *Brassica napus* [13]. Catechins, which belong to flavon-3-ols, are the major quality-related secondary metabolites with astringe taste and antioxidant activities in *Camellia sinensis* [L.] O. Kuntze.

Plant C2H2-ZFPs could enhance abiotic tolerance by enhancing the ability to scavenge reactive oxygen. Overexpressing *PeSTZ1* (a C2H2-type zinc finger transcription factor from *Populus euphratica*) in 84K poplar enhances freezing tolerance through the modulation of ROS scavenging via the direct regulation of *ASCORBATE PEROXIDASE2* expression [14]. Tomato SlZF3 directly binds CSN5B to promote the accumulation of ascorbic acid and increase ascorbic acid-mediated ROS-scavenging capacity, enhancing plant salt-stress tolerance [15]. OsZFP213 cooperates with OsMAPK3 in maintaining reactive oxygen species (ROS) homeostasis by enhancing the expression and activity of antioxidant enzymes for the regulation of salt-stress tolerance in rice [16]. Plant C2H2-ZFPs enhance stress resistance by directly regulating tolerance-related genes as well. A typical C2H2-ZFP gene *GmZF1* from soybean functions to increase the expression of the cold-responsive gene *cor6.6* to enhance the cold tolerance, probably by recognizing protein-DNA binding sites in *Arabidopsis* [17].

In addition, C2H2-ZFPs are involved in the tolerance response through the hormone-dependent pathway in plants. *AZF1* (At5g67450), *AZF2* (At3g19580) and *ZAT10* (At1g27730) are induced by drought and salt treatment through the ABA-dependent signaling pathway [18,19]. The EAR motif of C2H2-type zinc finger protein ZAT7 (At3g46090) directly inhibits WRKY70 expression during salinity stress in *Arabidopsis*, which is a node of convergence for jasmonate-mediated and salicylate-mediated signals in plant defense [20,21]. The potato C2H2-ZFP gene, *StZFP2*, is significantly repressed by JA and may be directly involved in the response to herbivory [22]. The expression of *C2H2-ZFPs* is under control of phytohormones; however, the feedback regulation is established with their effects on phytohormone biosynthetic genes. Previous studies showed that ZAT10 and AZF2 regulate the expression of the JA biosynthesis gene *LOX3* to repress the JA signaling in the JA response [23].

To date, the whole genome-wide analysis of C2H2-ZFPs in higher plants has been reported widely: 176 C2H2-ZFPs in *A. thaliana* [24], 301 C2H2-ZFPs in *Brassica rapa* [25], 195 C2H2-ZFPs in *Gossypium raimondii* [26], 112 C2H2-ZFPs in tomato [27], 129 C2H2-ZFPs in cucumber [28] and 79 Q-type C2H2-ZFPs in *Solanum tuberosum* [29], etc. However, C2H2-ZFPs have not been identified yet in *C. sinensis*, even though tea is one of the most popular beverages with significant economic value worldwide. The growth and development of tea plants are influenced by various abiotic stresses, including salt, drought, and cold stresses, which directly constrain the yield and quality of tea [30,31,32]. Therefore, we performed a comprehensive study on the identification, classification, feature searching and expression profiling of *C2H2-ZFPs* in *C. sinensis*. In the present study, 134 C2H2-ZFPs were identified based on the genome-wide alignment of *C. sinensis*. Phylogenetic analysis revealed that CsC2H2-ZFPs were classified into four clades with the reference of C2H2-ZFPs in *A. thaliana*. CsC2H2-ZFPs in the same clade shared similar gene features, motif characters and expression patterns. The evolutionary analysis showed that most of the *CsC2H2-ZFPs* were segmentally duplicated genes and under negative selection, which exerted an effect on gene family expansion. In addition, *CsC2H2-ZFPs* were found in response to multiple stresses, including drought, cold, salt stress, and methyl jasmonate (MeJA) treatment according to the public genome database. Furthermore, the correlation analysis of *CsC2H2-ZFPs* and catechins content provides new insight into the potential function of *CsC2H2-ZFP* genes in quality-related metabolite accumulation in *C. sinensis*. Our results provide the genome-wide information for further research on the function-characterization of CsC2H2-ZFPs and improving the breeding of high-quality tea plants.

## 2. Results and Discussion

### 2.1. Identification and Phylogenetic Analysis of C2H2-Zinc Finger (C2H2-ZFP) Gene Family in C. sinensis

To identify all the members of the C2H2-ZFP gene family in *C. sinensis*, the hidden Markov model (HMM) profiles of C2H2-ZFP domain (PF00096), C2H2-ZFP 4 (PF13894), C2H2-ZFP 6 (PF13912), C2H2-zinc finger protein 10 (PF18414), C2H2-ZFP 11 (PF16622) and C2H2-ZFP 12 (PF18658) from the Pfam database were used for searching *CsC2H2-ZFP* genes (http://pfam.xfam.org/, accessed on 1 December 2020). The *CsC2H2-ZFP* genes were identified through alignment against the reference genome sequence (e-value < 0.1) [33]. In addition, the candidate sequences missing the C2H2-zinc finger domain were removed according to a SMART (http://smart.emblheidelberg.de/, accessed on 1 December 2020) and CDD (https://www.ncbi.nlm.nih.gov/cdd, accessed on 1 December 2020) search. Finally, a total of 134 *C2H2-zinc finger* genes were identified in *C. sinensis* (Table S1). The molecular weight (MW) of CsC2H2-ZFPs ranged from 9.13 kDa (CSS0039482) to 164.49 kDa (CSS0018552) and the length of amino acid sequences varied from 81 (CSS0039482) to 1477 (CSS0006398). In addition, the isoelectric point (pI) values of the CsC2H2-ZFPs were between 4.55 and 10.78 with 67.91% of them being over 7.0. Except for the multi-subcellular localization, the subcellular location of the CsC2H2-ZFPs were predicted mainly in the nucleus (Table S1).

**Figure 1:**
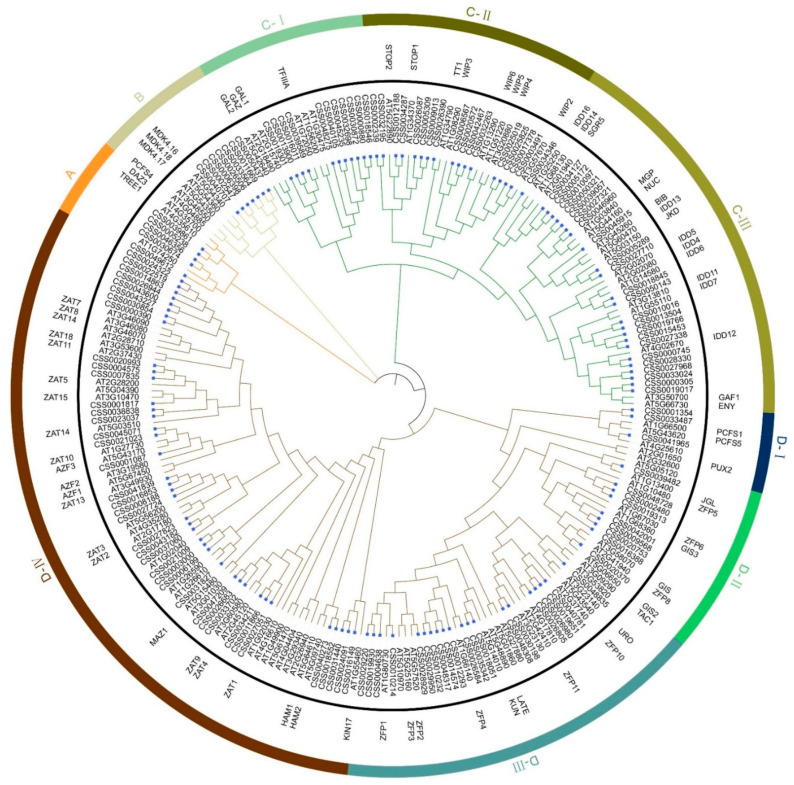
Phylogenetic classification of C2H2-Zinc Finger (C2H2-ZFP) proteins between *Arabidopsis thaliana* and *Camellia sinensis*. The phylogenetic tree was built with the maximum likelihood method for 1000 times bootstraps. Four subclasses were marked with arcs outside the circular tree using different colors. The blue rectangle represents CsC2H2-ZFPs from *C. sinensis*.

As the model dicotyledon *A. thaliana* C2H2-ZFPs have been extensively studied and many AtC2H2-ZFPs have been functionally characterized, a phylogenetic tree containing the C2H2-ZFPs from both *A. thaliana* and *C. sinensis* was constructed by the maximum likelihood method. All the CsC2H2-ZFPs were classified into four clades, namely clade A, clade B, clade C and clade D (Figure 1). Among them, clade C had the most members, followed by clade A and clade B. Clade A, B and C consisted of 5, 51 and 71 CsC2H2-ZFPs, respectively. Clade A contained the known TREE1 (At4g35610) and DAZ3 (At4g35700) which interact with EIN3 to inhibit shoot growth in response to ethylene [34]. Clade B had seven CsC2H2-ZFPs, including the PCFS4 (AT4G04885), which regulates *FCA* mRNA alternative processing to promote flowering in *Arabidopsis* [35]. Clade C was divided into three subclades—clade C-I, C-II and C-III. GAZ (AT2G18490), GAL1 (AT2G15740) and GAL2 (AT5G42640) in clade C-I have been reported to play a role in the transcriptional control of ABA and GA homeostasis during ground tissue maturation of the *Arabidopsis* root [36]. Clade C-II consisted of STOP and WIP-type C2H2-ZFPs. STOP1 (At1g34370) and STOP2 (At5g22890) play a critical role in the tolerance of major stress factors in acid soils [37,38]. WIP-type C2H2-ZFPs in *Arabidopsis*, such as TT1 (At1g34790), WIP2 (At3g57670) and WIP6 (At1g13290), have been reported to be involved in endothelium differentiation, normal differentiation of the ovary-transmitting tract cells and pollen tube growth, and leaf vasculature patterning [10,39]. The indeterminate-domain (IDD) type C2H2-ZFPs were clustered in clade C-III, such as IDD11 (At3g13810), IDD7 (At1g55110), IDD12 (At4g02670) and BIB(At3g45260). IDD-type C2H2-ZFPs have been implicated in diverse functions of plant metabolism and development, including root development, shoot gravitropism, leaf development, flowering time, seed maturation and hormone signaling, as well as the regulation of defense responses against hemibiotrophic pathogens [40].

In addition, there are four subclades in clade D. PUX2 was found in clade D-I, which acts as a negative regulator mediating the powdery mildew–plant interaction [41]. Clade D-II included GIS (At3g58070), ZFP8 (At2g41940), GIS2 (At5g06650), JGL (At1g13400), GIS3 (At1g68360), ZFP6 (At1g67030) and ZFP5 (At1g10480), contributing to trichome initiation and branching [5,42,43,44,45]. Most of *Arabidopsis* C2H2-ZFPs in clade D-III work as negative regulators of plant hormone signaling to regulate germination and early seedling, root and shoot development, such as ZFP1(At1g80730), ZFP3 (At5g25160), ZFP4 (At1g66140) [46]. In addition, the known AtC2H2-ZFPs in clade D-IV have been characterized to be involved in stress responses, while several AtC2H2-ZFPs mediate the regulation of male germ cell division and pollen fertility, such as MAZ1(At5g15480), DAZ1 (At2g17180) and DAZ2 (At4g35280) [47,48].

### 2.2. Distribution on Chromosome and Gene Duplication Events of C2H2-ZFP Gene Family in C. sinensis

**Figure 2:**
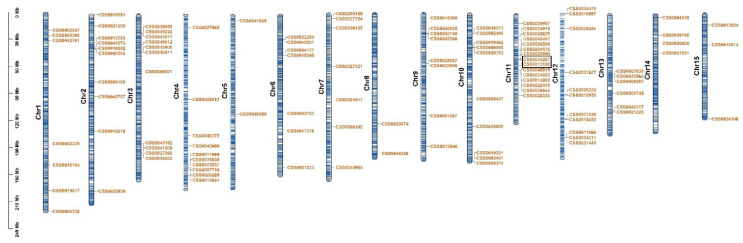
Chromosomal location of *CsC2H2-ZFP* genes in *C. sinensis* genome. The length of chromosomes is represented in Mb. Black lines within chromosomes indicate genes of *C. sinensis*. The number of chromosomes is presented at the left side of individual chromosomes. Genes of *CsC2H2-ZFP* are marked in red. Tandemly duplicated genes are marked with black boxes.

*CsC2H2-ZFPs* were distributed randomly on chromosomes in *C. sinensis*. Results showed that 113 *CsC2H2-ZFP* genes were distributed on 15 chromosomes in the *C. sinensis* genome while 21 *CsC2H2-ZFPs* were only assembled to contig and not presented on chromosomes (Figure 2). The location patterns of the *CsC2H2-ZFPs* were various across different chromosomes. Most of the *CsC2H2-ZFPs* genes on chromosome 11 were clustered at the centric region, while genes were predominantly distributed at the proximal region of chromosome 3 and genes were harbored randomly on chromosome 12. The numbers of *CsC2H2-ZFPs* ranged from 2 to 14. There were more genes on chromosomes 11 with 14 *CsC2H2-ZFP* genes and less on chromosome 5 and 8, each with two. In addition, the *CsC2H2-ZFPs* in the same clade in the phylogenetic tree clustered randomly on chromosomes.

**Figure 3:**
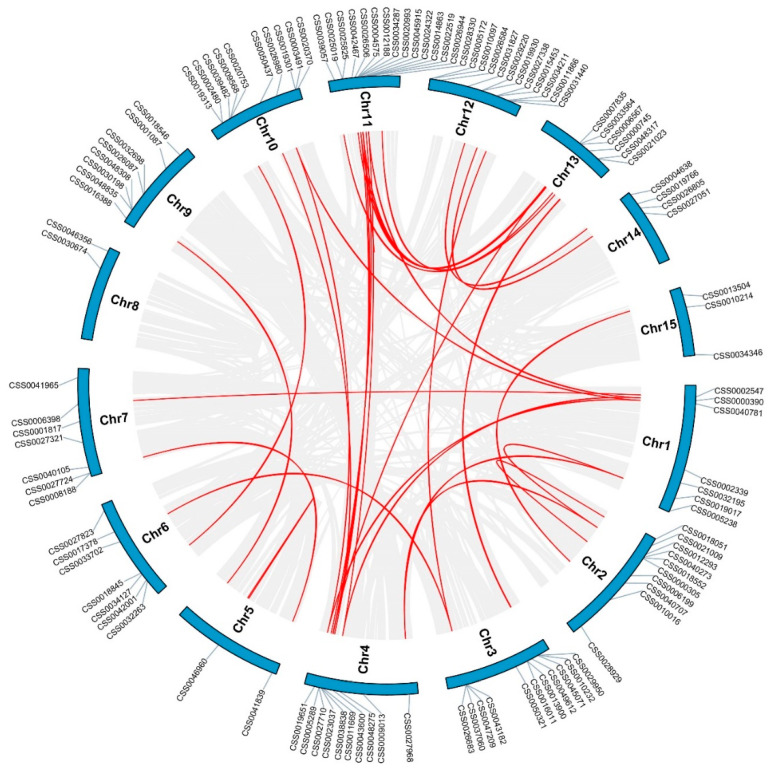
A syntenic relationship among *CsC2H2-ZFP* homologous genes was presented on the genome of *C. sinensis*. Gray lines indicate all synteny blocks and red lines indicate segmentally duplicated genes in *C. sinensis* genome. The chromosome number and gene names are presented.

Gene family expansion is caused by tandem gene duplication and segmental duplication. One pair of tandemly duplicated genes and 35 pairs of segmentally duplicated genes were identified in *C. sinensis*, respectively. Tandemly duplicated genes were found on chromosome 11 (Figure 2). The synteny map of the *C. sinensis* genome revealed that there were 36 pairs of homologs of *C2H2-ZFPs* in *C. sinensis* (Figure 3). These results indicate that the evolution of the CsC2H2-ZFPs gene family is coupled with gene duplication events and segmental duplication played a significant part in the expansion of the *CsC2H2-ZFP* genes. To further explore the divergence of *CsC2H2-ZFP* genes, the rate of nonsynonymous (Ka) and synonymous (Ks) substitution rates and Ka/Ks value of *CsC2H2-ZFPs* in *C. sinensis* were calculated (Table S2). The results showed that the Ka/Ks value of 34 pairs of *CsC2H2-ZFPs* was less than one, ranging from 0.1099 to 0.6413. Only two pairs orthologous genes (*CSS0011669* and *CSS0039057*, *CSS0011669* and *CSS0049612*) underwent positive selection (diversifying selection) with Ka/Ks value more than 1. These results indicate that most *CsC2H2-ZFP* genes experienced negative selection (purifying selection) in the process of evolution and have conserved characteristics at the protein level after the duplication events.

### 2.3. Gene Features and Conserved Motifs of the C2H2-ZFP Gene Family in C. sinensis

**Figure 4:**
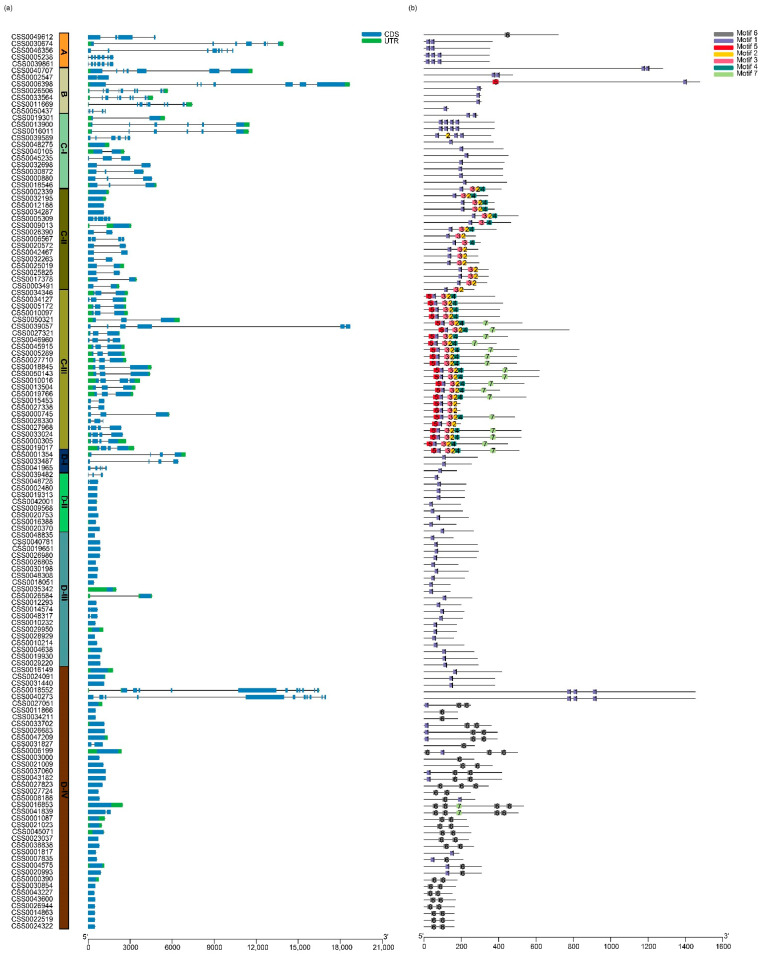
Gene structures and motif compositions of *CsC2H2-ZFP* genes. (**a**) The exon/intron structures of *CsC2H2-ZFPs* were predicted via TBtools. (**b**) The schematic representation of the conserved motifs as the colored box was identified by MEME web server. Intron is represented with a black line. The untranslated region (UTR) and coding sequence (CDS) are represented in colored boxes. The length of CsC2H2-ZFPs is indicated below.

The exon/intron organization of sequences reflects the structural diversity and complexity of *CsC2H2-ZFPs* (Figure 4). Our results show that 65 *CsC2H2-ZFPs* are intron-free, accounting for 48.5% of the total number of *CsC2H2-ZFPs*. By contrast, the intron number of the remaining *CsC2H2-ZFPs* ranges from 1 to 10. Paralogous pairs in the same phylogenetic tree clade share a common number and length of introns. *CsC2H2-ZFPs* in clade C show a diverse gene structure with great differences in their intron numbers, varying from 0 to 5. In clade D-IV, most sequences were intron-free, except for *CSS004189* and *CSS0031827*, with one intron, and *CSS0018552* and *CSS0040273*, with 10 introns.

Moreover, the motif compositions of CsC2H2-ZFPs were analyzed (Figure 4). Seven different kinds of motifs were identified in amino acid sequences of CsC2H2-ZFPs (Figure S1). Motif 1 was distributed in nearly all of the CsC2H2-ZFPs, which suggests that motif 1 could be a conserved motif among C2H2-ZFPs in *C. sinensis*. Motif 3 was only identified in clade C-II and C-III, motif 7 was mainly found in clade C-III, and motif 6 was mainly identified in clade D-IV, which implied that motifs 3, 6 and 7 were distributed in certain clusters of CsC2H2-ZFPs. Furthermore, the average density of motif in group B was less than that in clade C-II and clade C-III. These findings suggest that CsC2H2-ZFPs with various motifs are possibly associated with corresponding functions and might function divergently based on phylogenetic analysis.

### 2.4. Responses of CsC2H2-ZFP Genes under Stress

**Figure 5:**
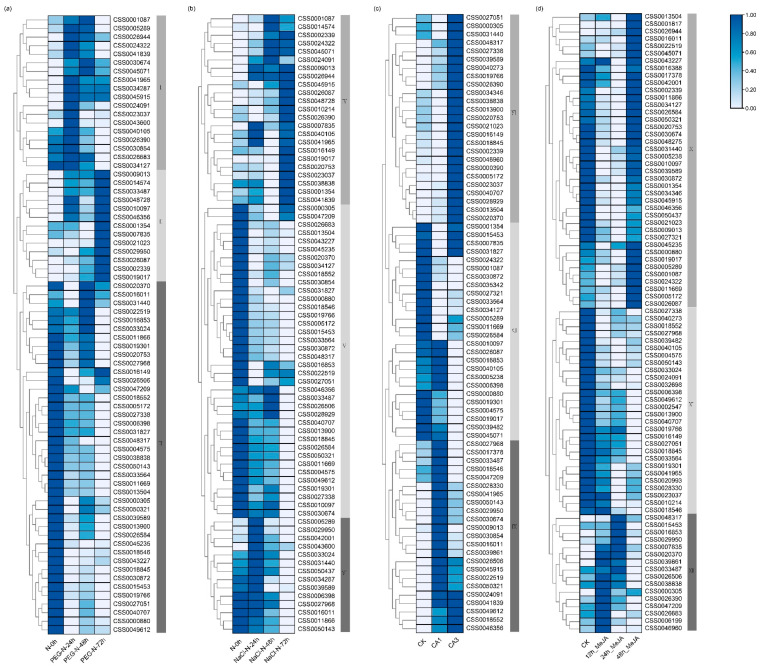
Expression patterns of *CsC2H2-ZFP* genes with different stresses. (**a**) Drought stress; (**b**) salt stress; (**c**) cold stress; (**d**) methyl jasmonate (MeJA) treatment. Control is placed at the leftmost column of heatmap. PEG, polyethylene glycol; CA1, fully acclimated; CA3, de-acclimated. The heatmap was visualized using log transformed values by TBtools software. The expression data from TIPA genome database was used to generate the heatmap by TBtools. The dark blue and light blue indicate high and low levels of gene expression, respectively.

A sudden drop in temperature, lack of water and soil salinization can cause severe damage or even death in *C. sinensis*. To better explore the expression patterns of *CsC2H2-ZFP* genes in response to abiotic stress, the expression level of *CsC2H2-ZFP* genes under cold, drought and salt stresses in cultivar Shuchazao (*C. sinensis* var. *sinensis*) were analyzed using the RNA-seq data published in the Tea Plant Information Archive (TPIA) database. Based on the expression patterns, the hierarchical clustering of 134 *CsC2H2-ZFPs* is shown in Figure 5. Under dehydration stress induced by 25% polyethylene glycol (PEG) treatment, all genes in group I and group II exhibited an upregulated trend, and the expression levels of genes in group III displayed a significantly downregulated trend. The transcriptional levels of *CSS0030872*, *CSS0050143*, *CSS0019766* and *CSS0011669* in group III was significantly downregulated by the drought stress, while the expression of *CSS0045071* in group I and *CSS0002339, CSS0009013, CSS0014574* in group II strongly upregulated after the drought stress (Figure 5a). Therefore, *CsC2H2-ZFPs* may be important in response to drought stress. Furthermore, the profile of these genes under the drought stress shared the same trend with that in the salt stress (Figure 5b), indicating that some *CsC2H2-ZFP* genes may be sensitive to abiotic stresses and their expression is induced under different kinds of abiotic stress. In addition, the expression of *CSS0048317*, *CSS0030854*, *CSS0040273*, *CSS0018552* and *CSS0020370* was upregulated in fully acclimated (CA1) and de-acclimated (CA3) cold stress.

MeJA could induce the accumulation of secondary metabolites, initiate a defense response upon mechanical damage or insect attack, and enhance the cold tolerance in *C. sinensis* [37,38]. Thus, the heatmap of the *CsC2H2-ZFP* gene expression level with MeJA treatment was performed (Figure 5c). *CsC2H2-ZFP* genes exhibited three patterns under the MeJA treatment including an early upward response at 12 to 24 h, late upward response at 48 h, and downward trend (Figure 5d). The expression of all 16 genes in group XII had an obvious up-regulation after 12 h or 24 h MeJA treatment, especially *CSS0039861*, *CSS0029950*, *CSS0016853*, *CSS0007835* and *CSS0020370* showing a high abundant gene expression. Genes in group X showed the highest gene expression in 48 h-MeJA treated samples, such as *CSS0001087*, *CSS0022519*, *CSS0045071*. *CsC2H2-ZFPs* in both group X and group XII were significantly induced by MeJA treatment, implying that they might be potential MeJA-responsive genes and might regulate the biosynthesis of plant specialized-metabolites and plant defense mechanisms. In addition, genes in group XI showed a downward trend and the highest gene expression was found in untreated samples. Taken together, these results reveal complicated and dynamic changes of MeJA-mediated *C2H2-ZFPs* gene expression.

### 2.5. Combined Analysis of Catechin Content and Expression of CsC2H2-ZFP Genes in Young Tissues in C. sinensis

**Figure 6:**
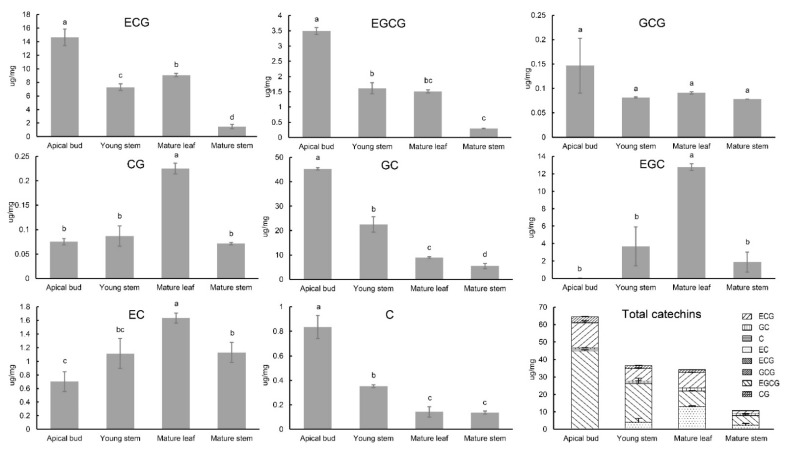
Catechins accumulation in different tissues of tea plants. C, catechin; EC, epicatechin; CG, catechin gallate; GC, gallocatechin; EGC, epigallocatechin; ECG, epicatechin gallate; EGCG, epigallocatechin gallate; GCG, gallocatechin gallate. Total catechins were calculated by the sum of all above seven catechins content. Statistically significant differences based on Student’s *t*-test, *p* < 0.05 are indicated by letters. And the same letter means insignificant changes, while the different letters between two bars mean significant change. Error bar is standard error. Each data contained three independently biological replicates.

In the tea processing industry, the high-quality tea beverage is mainly produced with apical buds and young tissues while the commercial tea bags use mature leaves, stems or branches as ingredients, which reflects a huge difference in the taste and health-protecting components between them. In order to explore the putative role of *CsC2H2-ZFP* genes in tea quality, apical buds, mature leaves, young stems and mature stems were obtained from tea plants, and transcriptome analysis combined with the catechins accumulation by HPLC was performed. Eight catechins, including catechin (C), epicatechin (EC), gallocatechin (GC), catechin gallate (CG), epigallocatechin (EGC), epicatechin gallate (ECG), epigallocatechin gallate (EGCG) and gallocatechin gallate (GCG), were detected in these tissues by HPLC (Figure 6). The highest level of the total content of the catechins was detected in apical buds. The results show that the content of GC is the highest among the rest of others while only a small amount of CG and GCG content was detected. Significantly, the total content of GC is approximately 200 times higher than that of GCG. The content of GC, EGC and C is higher than their esterified forms, but the opposite phenomenon occurred for EC and ECG. The ranking order of esterified and non-esterified catechins is listed as follows based on their total content in four different tissues: ECG > EGCG > CG > GCG, GC > EGC > EC > C. In addition, the distribution of tea catechins is tissue-dependent and the accumulation patterns of tea catechins are classified into three categories. ECG, EGCG, GCG, GC and C mainly accumulated in apical buds while CG, EGC and EC are preferentially distributed in mature leaves rather than in apical buds or young stems. Buds and leaves contain a higher abundance of catechins than stems, in terms of ECG, EGCG, GCG, GC and C. The kinetic change of ECG, EGCG, GC and C shares a common trend that the younger the tea tissues are, the relatively higher content they contain compared with the mature ones.

The total catechins content varies in different tissues and the content of individual catechin monomers are variable in each tissue. It has been reported that the total catechins are mainly distributed in young leaves in tea plants [49,50], which is consistent with our results. As for the catechin monomer, previous studies showed different metabolic profiles with leaf development in different cultivars. The levels of EGCG, EGC and ECG were high in young leaves compared to those in the mature leaves in tea strain 1005, while the levels of EC were the opposite [50]. The content of catechin monomers was significantly high in shoots in contrast to mature leaves, except the insignificant differences of GC accumulation in Oolong No. 17 [49]. In our results, most of the catechin monomers showed the highest accumulation in young leaves in the Jinxuan cultivar, except CG, EGC and EC. Furthermore, significant differences in the catechins concentration were identified in eight tea plant cultivars, indicating that different cultivars affect catechins accumulation patterns as well [51]. In addition, many factors might affect the catechins accumulation, including tea harvest seasons and tea plant age.

**Figure 7:**
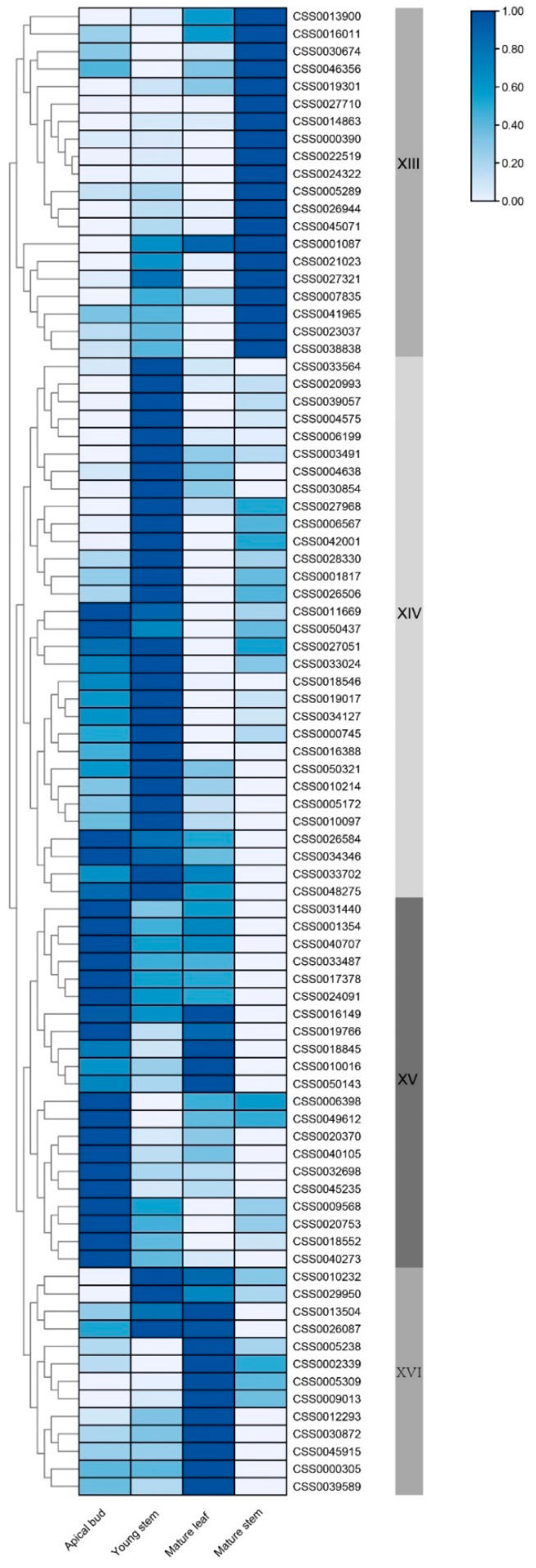
Expression profile of *CsC2H2-ZFP* in different tissues. The heatmap was visualized using log transformed values by TBtools software. The expression data from RNA-seq was used to generate the heatmap by TBtools. The dark blue and light blue indicate high and low level of gene expression, respectively.

The results of our in-house RNAseq data showed that 10 genes in group XV had the specific gene expression in apical buds, including *CSS0020370*, *CSS0006398*, *CSS0049612*, *CSS0040105*, *CSS0032698*, *CSS0045235*, *CSS0009568*, *CSS0020753*, *CSS0018552* and *CSS0040273* (Figure 7). The remaining genes in group XV showed high expression in both apical buds and young stems. In addition, 14 *CsC2H2-ZFP* genes in group XIV showed specific gene expression in young stems. These results indicate that *CsC2H2-ZFPs* had different expression patterns in the young tissues, suggesting that they may be involved in quality-related metabolite accumulation in young tissues in *C. sinensis*.

**Figure 8:**
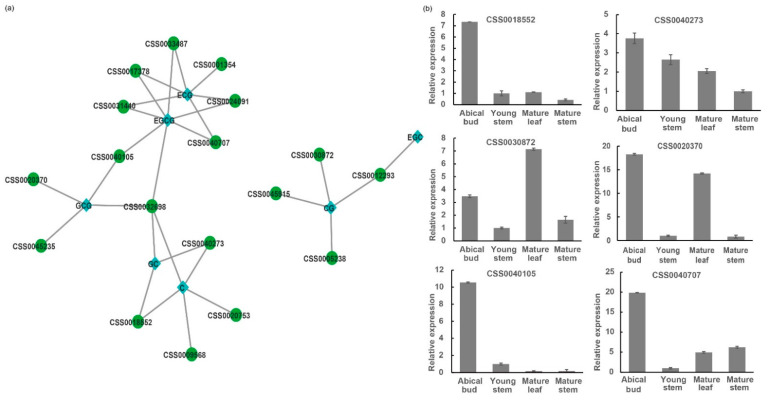
Correlation analysis and gene expression validation of catechins accumulation-related *CsC2H2-ZFPs*. (**a**), correlation network of *CsC2H2-ZFP* genes and catechins content; (**b**), Relative gene expression of candidate *CsC2H2-ZFPs* by qRT-PCR. The node color indicates the different metabolites or genes in the figure. The green nodes are *CsC2H2-ZFP* genes and blue nodes are metabolites. *CsC2H2-ZFPs* and catechins correlations with R^2^ > 0.9 are represented as links between nodes (*p* < 0.05). *18s rRNA* was used as the internal control for normalization and relative expression levels were calculated using the 2^(−^^∆∆Ct)^ method; error bars represent the standard deviations from three biological replicates.

To elucidate the functional relationship and identify new regulatory factors for catechins accumulation in *C. sinensis*, a gene-metabolite correlation network has been performed using Pearson correlation coefficients. The correlation analysis of *CsC2H2-ZFPs* expression and catechins content showed that 18 *CsC2H2-ZFPs* significantly correlated with six catechins (R^2^ > 0.9, *p* < 0.05). There was a significant correlation between GC and *CSS0032698*, *CSS0018552* and *CSS0040273* (Figure 8, Table S3). GCG was significantly correlated with *CSS0020370*, *CSS0032698*, *CSS0045235* and *CSS0040105*. Both ECG and EGCG were correlated with *CSS0017378, CSS0031440, CSS0040707, CSS033487* and *CSS0024091*. *CSS0032698* showed a significant correlation with GCG, GC and EGCG. These results reveal that specific *CsC2H2-ZFPs* were highly correlated with catechins content, indicating that these genes might play a vital role in catechins accumulation.

To validate the obtained expression data, six *CsC2H2-ZFPs* (*CSS018552*, *CSS0020370*, *CSS0030872*, *CSS0040105*, *CSS0040273*, *CSS0040707*) were selected for confirmation by qRT-PCR in the four tissues. The expression levels of the six genes were basically consistent with the RNA-seq results. Furthermore, the results showed that the transcription levels of *CSS018552* and *CSS0040273* shared similar profiles with the GC and C accumulation in different tissues (Figure 6, Figure 8). The expression level of *CSS0020370* was highly correlated with the GCG accumulation. *CSS030872* showed the highest gene expression in mature leaves, which is consistent with the high CG content in mature leaves (Figure 6). The transcription levels of *CSS0040105* and *CSS0040707* were highest in the apical bud, which match with the metabolic profiles of EGCG and ECG. Of them, *CSS0020370* responded to MeJA treatment in 12 h and 24 h (Figure 5d) and showed a high correlation with GCG (Figure 8), indicating the putative JA-regulated manner for the catechins biosynthesis and accumulation.

## 3. Materials and Methods

### 3.1. Identification and Characteristics of the C2H2-ZFP Gene Family in C. sinensis

The genomic library, cDNA library and protein database of *C. sinensis* were obtained from the Tea Plant Information Archive (TPIA, http://tpia.teaplant.org/index.html, accessed on 1 December 2020) database. The HMM profiles of the ZFP domains including the zinc finger protein (ZFP) domain (PF00096), C2H2-ZFP 4 (PF13894), C2H2-ZFP 6 (PF13912), C2H2-ZFP 10 (PF18414), C2H2-ZFP 11 (PF16622) and C2H2-ZFP 12 (PF18658) were downloaded from the Pfam website (http://pfam.xfam.org/, accessed on 1 December 2020). All *C2H2-ZFP* genes were identified based on the HMM profiles of the above domains from Pfam by using TBtools [52]. Then, the ZFP domain was checked by the Modular Architecture Research Tool (SMART, http://smart.embl-heidelberg.de/, accessed on 1 December 2020) and NCBI Conserved Domain Data (CDD, https://www.ncbi.nlm.nih.gov/cdd, accessed on 1 December 2020) to confirm all the *C2H2-ZFP* genes containing at least one ZFP domain. Genes missing the ZFP domains were removed from candidates.

### 3.2. Chromosomal Location of C2H2-ZFP Gene Family in C. sinensis

The position information of C2H2-ZFP family genes was acquired by TPIA database. The gene chromosomal location and distribution were visualized by TBtools and MCScanX, respectively [52,53]. Gene duplication events of the *C2H2-ZFP* genes were confirmed based on their copy number and genomic distribution. The default e-value cutoff of MCScanX is 1 × e^−10^. In addition, two genes located in the same chromosomal fragment within 100 kb and separated by five or fewer genes were identified as tandemly duplicated genes. The Ka and Ks were calculated by counting the numbers of synonymous and nonsynonymous sites and calculating the numbers of synonymous and nonsynonymous substitutions [54]. A Ka/Ks value more or less than 1 implies the occurrence of positive selection (also called diversifying selection) and negative selection (also called purifying selection), respectively, while if it equals 1, means neutral selection.

### 3.3. Phylogenetic Analysis of C2H2-ZFP Gene Family in C. sinensis

The full-length amino acid sequences of *C. sinensis* and *Arabidopsis* C2H2-ZFPs were used for phylogeny analysis. The amino acid sequences were aligned by MAFFT and the maximum likelihood phylogenies were inferred using FastTree with the Jones–Taylor–Thornton model for 1000 times bootstraps [55]. Furthermore, the phylogenetic tree was visualized using iTOL (https://itol.embl.de/, accessed on 8 January 2021).

### 3.4. Exon/Intron Structure Analysis and Identification of Conserved Motifs

The exon/intron structure of *CsC2H2-ZFPs* was analyzed by TBtools. The conserved motifs of CsC2H2-ZFPs were predicted by the Multiple Expectation Maximization for Motif Elicitation (MEME, https://meme-suite.org/meme/, accessed on 28 December 2020) online server. The subcellular localization of CsC2H2-ZFPs was predicted by WoLF PSORT (https://wolfpsort.hgc.jp/, accessed on 28 December 2020).

### 3.5. Plant Materials

Samples were collected from 12-year-old tea plants Jinxuan (*Camellia sinensis* var. *sinensis*) grown in the Tea Experiment Field of the South China Agricultural University, Guangzhou, China in spring. Tea leaves and stems were named from top to bottom, which are the apical bud, mature leaf, young stem and mature stem. The fourth leaves were collected as mature leaves. Similarly, the first and second sections of stems were harvested as young stem, and the fourth and fifth sections of stems were used as mature stem. Each sample was pooled from ten individual tea plants, and a total of 30 plants were used to form three pooled replications. The samples were immediately frozen in liquid nitrogen and stored at −80 °C for transcriptomic and phytochemical analysis.

### 3.6. Expression Analysis of the C2H2-ZFP Gene Family in C. sinensis

RNA-seq data of *C. sinensis* cultivar Shuchazao under treatments of cold, drought, salt stress and MeJA were retrieved from TPIA database. In addition, our in-house RNA-seq data of four tissues (apical bud, mature leaf, young stem and mature stem) in cultivar Jinxuan was sequenced on an Illumina platform and paired-end reads were generated at Biomarker Technology Services (Beijing, China). Due to cultivar Shuchazao and Jinxuan belonging to the diploid of *C. sinensis* var. *sinensis*, clean reads were mapped to the updated chromosome-leveled tea genome of cultivar Shuchazao [56].

The gene function was annotated based on the following databases: Nr (NCBI non-redundant protein sequences), Nt (NCBI non-redundant nucleotide sequences), Pfam (protein family), KOG/COG (clusters of orthologous groups of proteins), Swiss-Prot (a manually annotated and reviewed protein sequence database), KO (KEGG Ortholog database) and GO (Gene Ontology). StringTie was run to estimate the expression level using a maximum flow algorithm, and FPKM (fragments per kilobase of transcript per million fragments mapped) values were measured as the transcript expression levels. The heatmap of *CsC2H2-ZFPs* gene expression profiles were generated using the TBtools.

### 3.7. HPLC Analysis of Catechins

Tea samples (100 mg, fresh weight) were extracted with 400 μL 75% (*v*/*v*) methanol. After a brief vortex, the samples were sonicated at 70 °C for 10 min. The extracts were centrifuged at 10,000× *g* at 4 °C for 10 min and the supernatant was filtered through 0.45 μm Millipore filters for HPLC analysis. The measurement of catechins was performed in a Shimadzu LC-16 system (Shimadzu, Kyoto, Japan), which was equipped with a reversed phase column (WondaSil C18-WR 5 μm, 4.6 mm × 150 mm), an auto-sampler (SIL-16, Shimadzu, Kyoto, Japan), the photodiode array detector (SPD-16, Shimadzu, Kyoto, Japan), and a binary pump (LC-16, Shimadzu, Kyoto, Japan). The mobile phase was composed of ultra-purified water with 0.1% (*v*/*v*) formic acid (A), and acetonitrile (B) with the following linear gradient elution: 0.0 to 5.0 min: 4.0 to 6.0% B; 5.0 to 25.0 min: 6.0 to 10.0% B; 25.0 to 49.0 min: 10.0 to 21.0% B; 49.0 to 52.0 min: 21.0% B; 52.0 to 52.5 min: 21.0 to 4.0% B; 52.5 to 54.0 min: 4.0% B. The column temperature was set at 40 °C. The samples were eluted at 1 mL min^−1^ flow rate and monitored at 280 nm. The catechin monomers were identified by their retention time of the commercial standards under the same HPLC conditions. The integrated peak area was used for the quantification and calibration curve by plotting the peak area against the concentration of each standard compound, obtained to determine the concentration of respective catechin monomers. Each sample was performed in three biological replicates. The Pearson correlation coefficient among genes and metabolites was calculated by SPSS21.0 with the threshold set as 0.9 and *p* < 0.05.

### 3.8. RNA Isolation and Quantitative Real-Time PCR Validation

Total RNA was isolated and purified according to previous methods using the RNAprep Pure Plant Kit (Tiangen, Beijing, China) [57]. To ensure the accuracy of the target genes, quantitative real-time PCR (qRT-PCR) analyses were carried out to validate the expression of various catechins biosynthetic genes. An aliquot of 1 µg of total RNA was converted to first-strand cDNA using a PrimeScript RT enzyme with a gDNA eraser (Takara, Japan). The CDS (coding sequence) generated from the reference genome was downloaded from TPIA database (NCBI accession number PRJNA597714) for the primer design. The primers for qRT-PCR were designed by Primer3Plus (https://primer3plus.com/, accessed on 20 January 2021) and listed in Table S4. The qRT-PCR was performed on an CFX96 Real-Time PCR Detection System (Bio-Rad, Foster City, CA, USA) using SYBR Premix Ex Taq II (Takara, Japan). Next, the transcript levels of the target genes were monitored with *18s rRNA* as the internal control for normalization and calculated using the 2^−∆∆ct^ method [58]. Moreover, ΔCt variation analyses at different template concentrations were performed for each primer pairs to validate the 2^−∆∆ct^ method, and the relative ΔCt equations were listed in Table S4 [58,59]. All these experiments were performed with three biological replicates.

## 4. Conclusions

C2H2-ZFPs are important regulatory factors that function in plant development, in response to stress, and even in late flavonoid accumulation. In this study, a total of 134 *C2H2-ZFPs* were identified in *C. sinensis*. These genes were distributed on 15 chromosomes. The major gene expansion of the C2H2-ZFP gene family in *C. sinensis* was segmental duplication and under negative selection. In addition, *CsC2H2-ZFPs* were found in response to multiple stresses, including drought, cold, salt stress and MeJA treatment according to the public genome database. Notably, several sets of CsC2H2-ZFPs showed a significant correlation with the catechins content, which might contribute to the tea quality and specialized astringent taste. These results suggest that CsC2H2-ZFPs might play critical roles in tea quality and taste formation, which will provide new insight into both the stress responses and quality breeding in *C. sinensis*.

## Data Availability

The metabolomics data used to support the findings of this study are included within the Supplementary Information files.

## Supplementary Materials

The following are available online at https://www.mdpi.com/article/10.3390/ijms22084197/s1, Table S1: Prediction of subcellular localization and the properties of identified C2H2-ZFPs in *C. sinensis*. Table S2: Synonymous (Ks) and non-synonymous (Ka) substitution rates are represented for each gene pairs of *CsC2H2-ZFPs*. Table S3: Correlation coefficients between *CsC2H2-ZFPs* expression level and catechins content based on Pearson’s correlation analysis. Table S4: Sequences of primers used in qRT-PCR. Figure S1: The motif sequences of C2H2-ZFPs in *C. sinensis*.
