# Supplementary material for: Genome-Wide Identification and Expression Patterns of the C2H2-Zinc Finger Gene Family Related to Stress Responses and Catechins Accumulation in Camellia sinensis [L.] O. Kuntze
Source: Int J Mol Sci. 2021 Apr 18;22(8):4197. doi: 10.3390/ijms22084197 (PMC8074030; doi:10.3390/ijms22084197)

Table S1. Prediction of subcellular localization and the properties of identified *C2H2-ZFPs* in *C. sinensis.*

Gene ID	Subcellular Localization site	Length of amino acids	molecular weight (kDa)	Theoretical pI
CSS0000305	nucl: 14	448	48.15	8.80
CSS0000390	nucl: 14	178	19.33	9.10
CSS0000745	nucl: 13.5, cyto_nucl: 7.5	485	53.85	9.07
CSS0000880	nucl: 14	422	45.39	9.22
CSS0001087	nucl: 14	228	24.33	8.78
CSS0001354	nucl: 13, pero: 1	287	32.36	8.81
CSS0001817	nucl: 14	188	20.53	6.50
CSS0002339	nucl: 14	413	45.89	5.78
CSS0002480	nucl: 14	216	25.01	8.17
CSS0002547	nucl: 14	475	53.98	8.53
CSS0003000	nucl: 14	268	30.43	5.20
CSS0003491	nucl: 13.5, cyto_nucl: 7.5	268	29.98	6.45
CSS0004575	nucl: 14	307	33.61	5.97
CSS0004638	nucl: 13, pero: 1	268	29.34	6.79
CSS0005172	nucl: 13.5, cyto_nucl: 7.5	406	45.35	8.57
CSS0005238	nucl: 12, cyto: 1, pero: 1	350	40.46	8.39
CSS0005289	nucl: 14	497	53.78	9.23
CSS0005309	nucl: 13, pero: 1	465	52.34	5.53
CSS0006199	nucl: 14	501	55.01	8.84
CSS0006398	nucl: 12, extr: 2	1477	163.68	6.51
CSS0006567	nucl: 14	301	34.79	7.94
CSS0007835	nucl: 14	208	23.01	7.08
CSS0008188	nucl: 14	273	29.59	5.51
CSS0009013	nucl: 11.5, cyto_nucl: 6.5, extr: 2	386	42.31	6.31
CSS0009568	nucl: 13, pero: 1	207	21.67	7.26
CSS0010016	nucl: 14	536	58.02	8.72
CSS0010097	nucl: 13.5, cyto_nucl: 7.5	406	45.33	8.60
CSS0010214	nucl: 14	214	23.64	9.13
CSS0010232	nucl: 14	175	19.58	9.56
CSS0011669	nucl: 14	306	33.58	5.17
CSS0011866	nucl: 13, pero: 1	179	19.97	5.74
CSS0012188	nucl: 11.5, cyto_nucl: 6.5, extr: 1, pero: 1	376	42.18	8.85
CSS0012293	nucl: 14	198	22.27	7.82
CSS0013504	nucl: 14	406	44.42	9.04
CSS0013900	nucl: 14	376	43.07	5.97
CSS0014574	nucl: 14	214	23.87	10.78
CSS0014863	nucl: 14	161	17.73	9.75
CSS0015453	nucl: 14	194	21.99	9.68
CSS0016011	nucl: 14	376	43.07	5.97
CSS0016149	nucl: 12, chlo: 2	415	48.04	6.59

CSS0016388	nucl: 14	172	19.11	6.39
CSS0016853	nucl: 14	534	59.06	7.27
CSS0017378	nucl: 14	336	37.30	8.42
CSS0018051	nucl: 13, pero: 1	141	15.31	8.57
CSS0018546	nucl: 12, extr: 2	442	47.83	9.35
CSS0018552	nucl: 14	1453	164.49	6.30
CSS0018845	nucl: 13.5, cyto_nucl: 7.5	617	67.19	9.02
CSS0019017	nucl: 14	508	53.24	8.46
CSS0019301	nucl: 14	289	31.93	8.56
CSS0019313	nucl: 14	216	24.85	8.70
CSS0019651	nucl: 14	291	32.84	7.23
CSS0019766	nucl: 14	546	60.12	8.69
CSS0019930	nucl: 13, pero: 1	285	31.59	7.72
CSS0020370	nucl: 14	265	29.30	6.61
CSS0020572	nucl: 14	288	32.93	9.02
CSS0020753	nucl: 13, pero: 1	238	25.33	7.17
CSS0020993	nucl: 14	307	33.61	5.97
CSS0021009	nucl: 13, extr: 1	366	40.84	4.89
CSS0021023	nucl: 14	239	25.66	7.69
CSS0022519	nucl: 11, chlo: 1, extr: 1, pero: 1	161	17.59	10.04
CSS0023037	nucl: 14	239	25.60	6.20
CSS0024091	nucl: 11, chlo: 1, cyto: 1, extr: 1	380	44.02	9.03
CSS0024322	nucl: 11, chlo: 1, extr: 1, pero: 1	161	17.45	9.87
CSS0025019	nucl: 14	344	38.89	6.42
CSS0025825	nucl: 14	344	38.87	6.42
CSS0026087	nucl: 13, pero: 1	505	56.36	5.88
CSS0026390	nucl: 14	275	31.27	8.14
CSS0026506	nucl: 14	309	33.36	4.61
CSS0026584	nucl: 14	257	27.86	5.83
CSS0026683	nucl: 14	393	44.09	5.86
CSS0026805	nucl: 14	182	20.21	5.32
CSS0026944	nucl: 14	164	17.75	9.98
CSS0026980	nucl: 14	280	31.77	6.73
CSS0027051	nucl: 14	249	27.75	9.02
CSS0027321	nucl: 13.5, cyto_nucl: 7.5	449	49.73	9.07
CSS0027338	nucl: 14	194	21.99	9.68
CSS0027710	nucl: 14	495	53.56	9.23
CSS0027724	nucl: 14	249	27.97	7.66
CSS0027823	nucl: 12.5, cyto_nucl: 7, chlo: 1	344	36.77	7.19
CSS0027968	nucl: 14	520	54.74	8.90
CSS0028330	nucl: 14	196	22.11	9.74
CSS0028929	nucl: 13.5, cyto_nucl: 7.5	158	17.49	8.69
CSS0029220	nucl: 14	290	32.11	8.18

CSS0029950	nucl: 14	175	19.55	9.56
CSS0030198	nucl: 14	237	26.36	8.43
CSS0030674	nucl: 14	366	39.37	8.25
CSS0030854	nucl: 14	170	18.49	9.90
CSS0030872	nucl: 11, extr: 2, chlo: 1	421	45.68	9.54
CSS0031440	nucl: 13, chlo: 1	380	44.05	8.86
CSS0031827	nucl: 14	271	30.63	7.68
CSS0032195	nucl: 14	342	38.23	6.50
CSS0032263	nucl: 14	295	33.16	5.86
CSS0032698	nucl: 14	430	46.70	9.48
CSS0033024	nucl: 14	520	54.80	8.81
CSS0033487	nucl: 13, pero: 1	253	28.68	8.49
CSS0033564	nucl: 14	305	33.40	4.55
CSS0033702	nucl: 14	361	40.79	6.27
CSS0034127	nucl: 14	422	47.07	8.74
CSS0034211	nucl: 13, pero: 1	179	19.97	5.74
CSS0034287	nucl: 11.5, cyto_nucl: 6.5, extr: 1, pero: 1	376	42.17	8.85
CSS0034346	nucl: 14	380	42.86	9.13
CSS0035342	nucl: 14	140	15.13	7.05
CSS0037060	nucl: 14	416	46.32	6.52
CSS0038838	nucl: 14	266	28.64	8.15
CSS0039057	nucl: 14	778	85.57	7.87
CSS0039482	nucl: 14	81	9.13	8.73
CSS0039589	nucl: 14	357	41.11	8.78
CSS0039861	nucl: 12, cyto: 1, pero: 1	350	40.48	8.39
CSS0040105	nucl: 14	424	45.83	9.45
CSS0040273	nucl: 14	1453	164.43	6.18
CSS0040707	nucl: 12, extr: 2	1278	141.90	8.78
CSS0040781	nucl: 14	285	32.47	6.37
CSS0041839	nucl: 14	505	56.11	8.25
CSS0041965	nucl: 13, pero: 1	175	19.98	8.63
CSS0042001	nucl: 13, pero: 1	196	20.33	7.86
CSS0042467	nucl: 14	288	32.93	9.02
CSS0043182	nucl: 14	416	46.32	6.52
CSS0043227	nucl: 14	151	16.55	9.25
CSS0043600	nucl: 14	168	18.67	9.04
CSS0045071	nucl: 14	252	26.59	7.68
CSS0045235	nucl: 13, chlo: 1	451	48.74	9.46
CSS0045915	nucl: 14	510	55.72	9.16
CSS0046356	nucl: 14	352	38.24	9.27
CSS0046960	nucl: 13.5, cyto_nucl: 7.5	388	42.95	9.11
CSS0047209	nucl: 14	393	44.11	5.90
CSS0048275	nucl: 11, extr: 2, chlo: 1	370	41.88	9.04

CSS0048308	nucl: 14	218	24.26	6.44
CSS0048317	nucl: 14	207	23.40	10.56
CSS0048728	nucl: 14	224	25.27	6.24
CSS0048835	nucl: 14	157	17.62	5.42
CSS0049612	nucl: 14	719	79.20	5.72
CSS0050143	nucl: 13.5, cyto_nucl: 7.5	615	66.96	9.02
CSS0050321	nucl: 13.5, cyto_nucl: 7.5	525	57.66	9.02
CSS0050437	nucl: 14	131	14.06	8.68

nucl: nucleus; mito: mitochondrial matrix; extr: extracellular; chlo: chloroplast thylakoid membrane; cyto: cytoplasm; cyto_nucl: cytoplasmic_nuclear.

Table S2. Synonymous (Ks) and non-synonymous (Ka) substitution rates are represented for each gene pairs of *CsC2H2-ZFPs*.

Gene 1	Gene 2	Ka	Ks	Ka/Ks	P-Value	Duplication type
CSS0012188	CSS0034287	0.0049	0.0097	0.5041	0.296308	tandem
CSS0026980	CSS0040781	0.6271	1.8719	0.3350	2.66E-09	segmental
CSS0000390	CSS0024322	0.3853	3.5064	0.1099	3.10E-16	segmental
CSS0000305	CSS0019017	0.1048	0.4821	0.2173	8.09E-25	segmental
CSS0019651	CSS0040781	0.5761	1.9808	0.2909	3.17E-11	segmental
CSS0000390	CSS0043600	0.4618	3.5315	0.1308	2.60E-22	segmental
CSS0019017	CSS0027968	0.2934	1.9836	0.1479	6.95E-45	segmental
CSS0002547	CSS0006398	0.1661	0.5104	0.3254	4.62E-15	segmental
CSS0019651	CSS0026980	0.1512	0.4820	0.3137	1.07E-08	segmental
CSS0011669	CSS0049612	2.1791	1.8195	1.1977	0.487406	segmental
CSS0009568	CSS0042001	0.1310	0.4515	0.2901	4.69E-09	segmental
CSS0000745	CSS0029220	1.7343	3.5763	0.4849	3.26E-05	segmental
CSS0006567	CSS0042467	0.1646	0.8894	0.1851	3.90E-18	segmental
CSS0026506	CSS0033564	0.1460	0.6582	0.2218	1.21E-14	segmental
CSS0007835	CSS0020993	0.2059	0.9136	0.2254	2.37E-13	segmental
CSS0011669	CSS0039057	3.5403	1.8400	1.9241	0.015522	segmental
CSS0011669	CSS0026506	0.4824	1.5190	0.3176	2.56E-13	segmental
CSS0004575	CSS0038838	0.4065	3.6667	0.1109	7.03E-25	segmental
CSS0020993	CSS0023037	0.4982	1.2425	0.4010	7.93E-07	segmental
CSS0005289	CSS0045915	0.3879	2.3455	0.1654	2.88E-28	segmental
CSS0024322	CSS0043600	0.2449	1.1722	0.2090	1.51E-11	segmental
CSS0014863	CSS0043227	0.2025	1.4241	0.1422	3.24E-14	segmental
CSS0015453	CSS0019766	0.0570	0.4110	0.1388	7.39E-14	segmental
CSS0004638	CSS0019930	0.1940	0.5754	0.3372	9.22E-09	segmental
CSS0034211	CSS0047209	0.4105	2.8433	0.1444	6.52E-15	segmental
CSS0010232	CSS0048317	0.1908	1.0683	0.1786	3.03E-12	segmental
CSS0021023	CSS0045071	0.1202	1.0357	0.1160	7.41E-24	segmental
CSS0007835	CSS0023037	0.3711	2.4106	0.1540	8.71E-18	segmental

CSS0010016	CSS0013504	0.1933	0.4861	0.3976	4.53E-09	segmental
CSS0006199	CSS0021009	1.9705	3.7688	0.5228	0.115405	segmental
CSS0000305	CSS0027968	0.3188	1.8877	0.1689	3.68E-45	segmental
CSS0033702	CSS0047209	0.1982	0.8849	0.2240	3.40E-21	segmental
CSS0039482	CSS0046960	1.5537	2.7921	0.5565	0.000179	segmental
CSS0008188	CSS0041839	1.5437	3.6197	0.4265	8.06E-19	segmental
CSS0015453	CSS0048835	2.1439	3.3432	0.6413	0.350698	segmental
CSS0018845	CSS0050143	0.0014	0.0046	0.3097	0.23652	segmental

Table S3. Correlation coefficients between *CsC2H2-ZFPs* expression level and catechins content based on Pearson's correlation analysis.

CsZFP	Catechins	R^2
CSS0040273	C	1.00**
CSS0018552	C	0.99**
CSS0032698	C	0.97*
CSS0020753	C	0.96*
CSS0009568	C	0.95*
CSS0012293	CG	0.98*
CSS0045915	CG	0.98*
CSS0030872	CG	0.97*
CSS0005238	CG	0.96*
CSS0001354	ECG	0.99**
CSS0040707	ECG	0.99**
CSS0031440	ECG	0.99*
CSS0017378	ECG	0.99*
CSS0033487	ECG	0.98*
CSS0024091	ECG	0.97*
CSS0012293	EGC	0.98*
CSS0033487	EGCG	1.00**
CSS0017378	EGCG	0.98*
CSS0024091	EGCG	0.97*
CSS0032698	EGCG	0.96*
CSS0040105	EGCG	0.96*
CSS0031440	EGCG	0.96*
CSS0040707	EGCG	0.95*
CSS0040273	GC	1.00**
CSS0018552	GC	0.98*
CSS0032698	GC	0.96*
CSS0045235	GCG	1.00*
CSS0020370	GCG	0.99*
CSS0040105	GCG	0.99*

CSS0032698	GCG	0.99*

* indicates significant correlation at the 0.05 level; ** indicates significant correlation at the 0.01 level.

Table S4. Sequences of primers used in qRT-PCR.

Gene name	Primer	Sequence （5’ to 3’）	Validation curves of the method by ΔCt variant analysis
CSS0018552	F	ACACCACGTGCAATTCGTTG	y = 0.002x + 10.113
	R	TTGCAGGATGAACCGAAAGC	
CSS0020370	F	TGTCCAAAATTCGCCTACGC	y = -0.0006x + 12.99
	R	ATCGGCGATGGCTTCAAATC	
CSS0030872	F	AGCTTCCACTGCAGCAATTC	y = -0.0005x + 11.858
	R	ATGCTTTCGCTTTGGCTTGG	
CSS0040105	F	TTCATGATCCGAAGTCCAGGAG	y = 0.0012x + 12.303
	R	TTGGGGGCTTTTCAATGTGC	
CSS0040273	F	AAGCTTTGGGCACGAAACAG	y = 0.0002x + 9.9693
	R	ACCACAACCCAACTGCAAAC	
CSS0040707	F	TGAGCTTTGCTTGTGTTGCC	y = 6E-05x + 11.773
	R	AGCCGCAGTTAAACCATCAC	

Figure S1 The motif sequences of C2H2-ZFPs in *C. sinensis.*

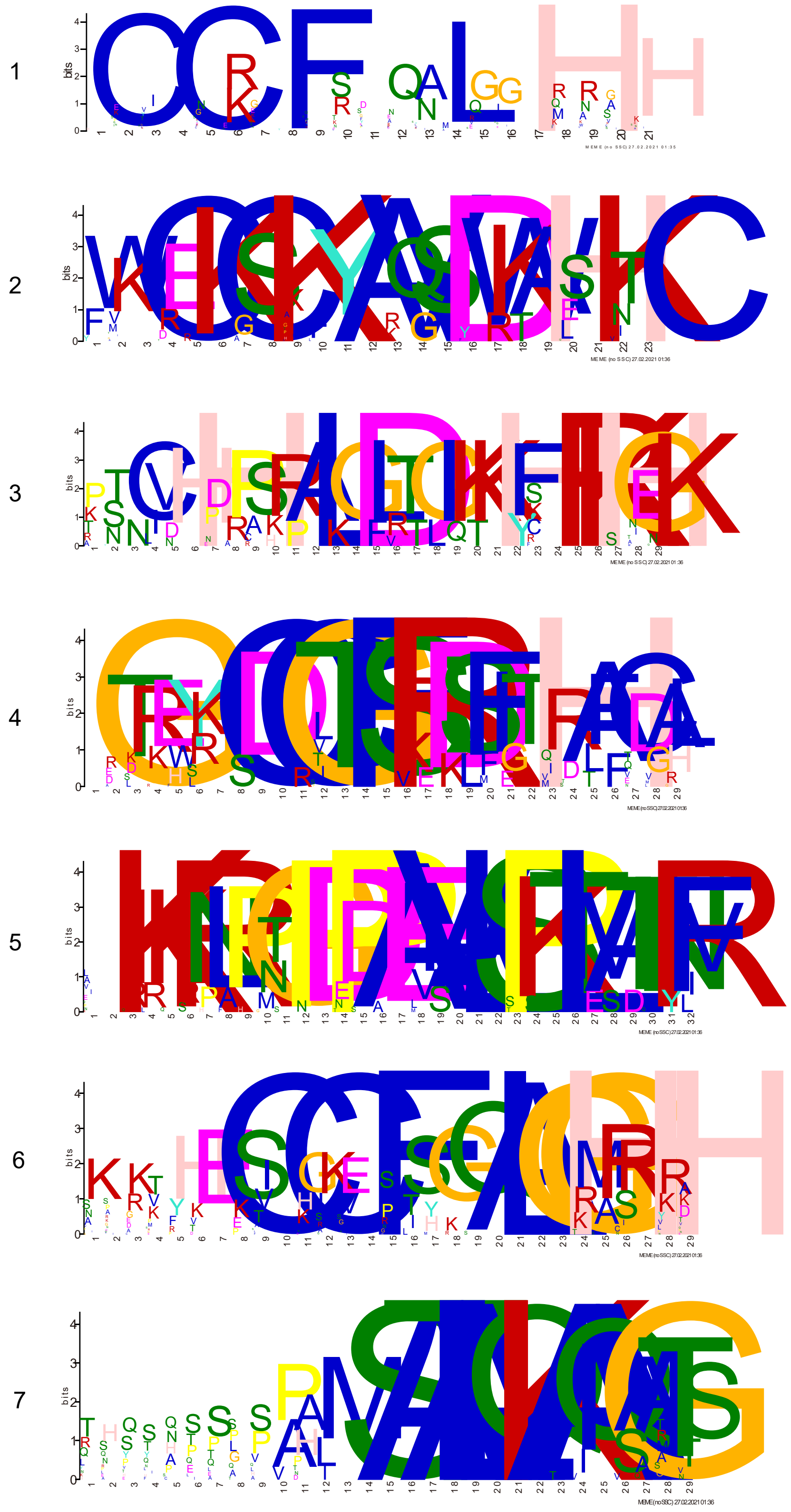

Supplement: Supplementary file 1 [file ijms-22-04197-s001.zip › ijms-1151505-supplementary.pdf]
